# First‐Line Osimertinib for *EGFR*‐Mutated Squamous Cell Lung Carcinoma: A Case Report

**DOI:** 10.1002/cnr2.70273

**Published:** 2025-07-07

**Authors:** Yugo Matsumura, Seiya Ichihara, Kaori Nii, Kazumasa Nanjo, Naoki Kadota, Yoshio Okano, Hisanori Machida, Nobuo Hatakeyama, Hiroyuki Hino, Keishi Naruse, Tsutomu Shinohara, Shoji Sakiyama, Eiji Tacheuchi

**Affiliations:** ^1^ Department of Respiratory Medicine National Hospital Organization Kochi Hospital Kochi Japan; ^2^ Department of Thoracic Surgery National Hospital Organization Kochi Hospital Kochi Japan; ^3^ Department of Pathology National Hospital Organization Kochi Hospital Kochi Japan; ^4^ Department of Community Medicine for Respirology Graduate School of Biomedical Sciences Tokushima Japan; ^5^ Department of Clinical Investigation National Hospital Organization Kochi Hospital Kochi Japan

**Keywords:** EGFR mutation, EGFR‐TKI, L858R, osimertinib, squamous cell lung cancer

## Abstract

**Background:**

Epidermal growth factor receptor (*EGFR*) mutations in squamous cell lung carcinoma are rare. EGFR‐tyrosine kinase inhibitors are generally less effective for *EGF*R‐mutated squamous cell lung carcinoma. We herein present a case of *EGFR*‐mutated squamous cell lung carcinoma that responded to osimertinib.

**Case:**

A 75‐year‐old woman with bloody sputum and left back pain was referred to NHO Kochi Hospital. A mass was observed in the left lower lobe on chest CT. Squamous cell lung carcinoma, cT4N1M1b (adrenal metastasis) stage IVA, was diagnosed based on the findings of a CT‐guided percutaneous lung biopsy and a CT scan revealing right adrenal metastasis. The primary tumor was subjected to a genomic analysis with the AmoyDx Pan Lung Cancer PCR panel, which revealed an *EGFR* mutation (exon 21 L858R). The PD‐L1 tumor proportion score was 95%. Osimertinib was initiated as first‐line targeted therapy. Tumor shrinkage was observed and maintained over 9 months of treatment.

**Conclusion:**

We encountered a rare *EGFR*‐mutated squamous cell lung carcinoma that responded well to osimertinib. Osimertinib may be an option for the treatment of patients with *EGFR*‐mutated squamous cell lung carcinoma.

## 1 Introduction

Squamous cell lung carcinoma is characterized by complex genomic abnormalities with an average of 360 exonic mutations, 165 genomic rearrangements, and 323 copy number alteration segments per tumor. Eleven mutated genes have been identified [1]. *TP53* mutations were found in most specimens; loss‐of‐function mutations in *HLA‐A* class I major histocompatibility genes have been reported [1]. Significantly affected pathways were *NFE2L2* and *KEAP1* in 34% of cases, squamous differentiation genes in 44%, phosphatidylinositol‐3‐OH kinase pathway genes in 47%, and *CDKN2A* and *RB1* in 72% [1].

Epidermal growth factor receptor (EGFR) is expressed in many solid tumors, including non‐small cell lung cancer, and is the origin of cancer growth signaling. *EGFR* mutations are common in Asian individuals, women, those with no history of smoking, and patients with adenocarcinoma [2, 3]. *EGFR* mutations have been reported in 47.9% of adenocarcinomas and 4.6% of squamous cell carcinomas in Asian populations, and 19.2% of adenocarcinomas and 3.3% of squamous cell carcinomas in Western populations, and are rare in squamous cell carcinomas [4]. The reported rate of *EGFR* mutations in patients with squamous cell carcinoma of the lung is 4.2%–7% [5, 6, 7, 8].

Although the efficacy of EGFR‐tyrosine kinase inhibitors (EGFR‐TKI) for *EGFR* mutation‐positive lung adenocarcinoma has been demonstrated, their effectiveness for *EGFR*‐mutated squamous cell carcinoma remains unclear. A retrospective study showed that first‐generation EGFR‐TKIs were less effective for *EGFR*‐mutated squamous cell lung carcinoma than for lung adenocarcinoma [9]. However, few studies have examined the efficacy of third‐generation EGFR‐TKIs.

We herein report a rare *EGFR*‐mutated squamous cell lung carcinoma that responded to osimertinib, a third‐generation EGFR‐TKI.

## 2 Case Report

**FIGURE 1:**
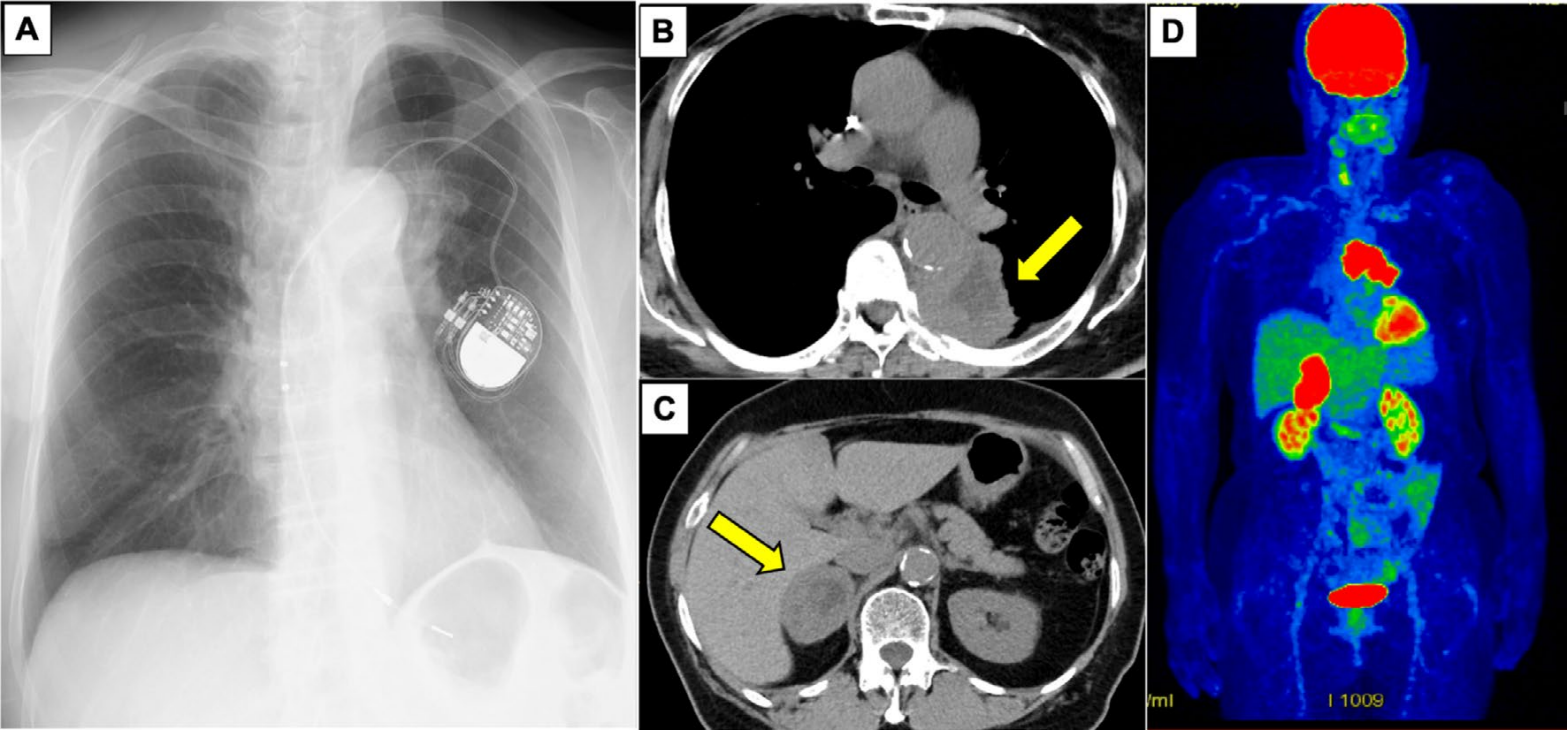
(A) Chest X‐ray image in the first visit to NHO Kochi Hospital in August 2023. A pacemaker was implanted and a mass shadow was present on the mediastinal side of the left lung field. (B and C) Computed tomography (CT) images taken during the first visit to NHO Kochi Hospital in August 2023. CT showed a mass shadow in the left lower lobe and right adrenal gland enlargement. (D) Positron emission tomography‐CT shows ^18^F‐fluorodeoxyglucose (FDG) uptake (the maximum standardized uptake value [SUVmax = 14.4]) in the mass of the left lower lobe and FDG uptake (SUVmax = 13.7) in the right adrenal gland.

A 75‐year‐old female without a history of smoking developed left back pain and bloody sputum and was referred to NHO Kochi Hospital for evaluation and treatment in August 2023. She had previously undergone surgery for breast cancer as well as the implantation of a pacemaker for a complete atrioventricular block. Chest X‐ray showed a mass on the mediastinal side of the left upper lung field and the pacemaker on the same side (Figure 1A). Chest and abdominal computed tomography (CT) revealed a 30‐mm mass in the left lower lobe (Figure 1B) and a 50‐mm mass in the right adrenal gland (Figure 1C).

On admission, she had left back pain and bloody sputum. Her score was 0 on the Eastern Cooperative Oncology Group performance status scale. A physical examination revealed no audible rales on lung auscultation or palpable peripheral lymph nodes. Her serum carcinoembryonic antigen (CEA) level was within the normal range. Serum cytokeratin 19 fragment and squamous cell carcinoma antigen levels were elevated at 21 and 3.2 ng/mL, respectively.

**FIGURE 2:**
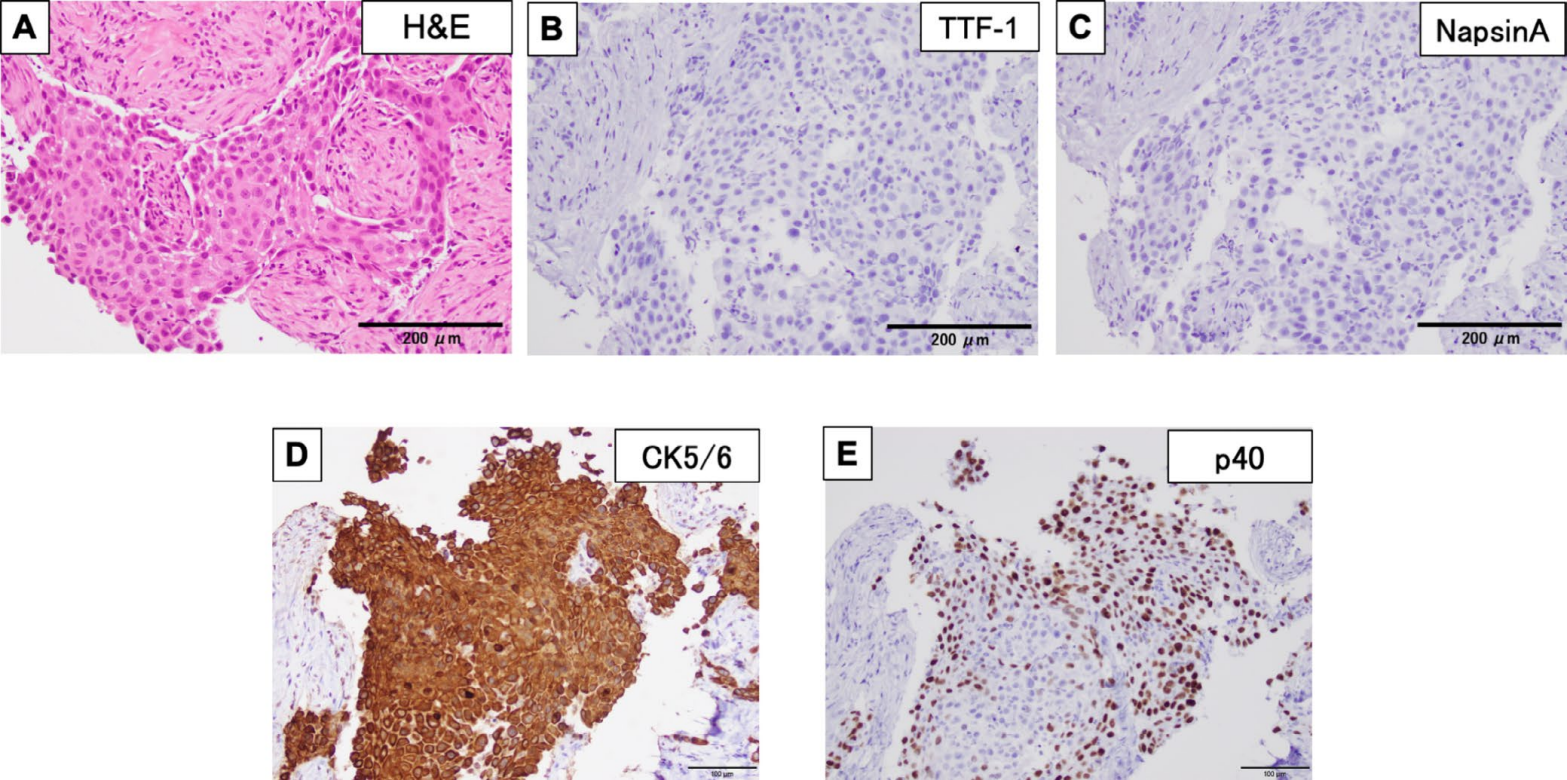
Histopathological findings of CT‐guided percutaneous lung biopsy. Pathological specimens stained with hematoxylin and eosin (H&E) showed the infiltration of tumor cells with intracellular bridges and their growth in sheets (A) (magnification: 100×; scale bar: 200 μm). Immunostaining revealed that tumor cells are negative for TTF‐1 (B) and NapsinA (C) (magnification: 100×; scale bar: 200 μm). Immunostaining revealed that the tumor cells were strongly positive for CK5/6 (D) and positive for p40 (E) (magnification: 200×; scale bar: 100 μm).

CT‐guided percutaneous lung biopsy was performed. The patient developed a fever after the CT‐guided percutaneous lung biopsy, which resolved with antibiotics. The histopathological examination of hematoxylin–eosin‐stained slides showed no keratinization; however, intercellular bridges were detected. Immunohistochemistry was negative for thyroid transcription factor‐1 (TTF‐1) and NapsinA, strongly positive for CK5/6, and positive for p40 (Figure 2). Based on these findings, a diagnosis of squamous cell carcinoma was made. The primary tumor was subjected to a genomic analysis with the AmoyDx Pan Lung Cancer PCR panel, which revealed an *EGFR* mutation (exon 21 L858R). Immunostaining showed that 95% of tumor cells expressed programmed death ligand 1 (22C3 clones). Brain metastatic lesions were not observed on contrast‐enhanced magnetic resonance images of the head. The uptake of ^18^F‐fluorodeoxyglucose (FDG) in the lung lesion (maximum standardized uptake value [SUVmax] = 14.4) and its accumulation in the right adrenal gland (SUVmax = 13.7) were noted on positron emission tomography‐CT (Figure 1D). Based on these findings, the patient was diagnosed with cT4N1M1b (adrenal metastasis) stage IVA.

**FIGURE 3:**
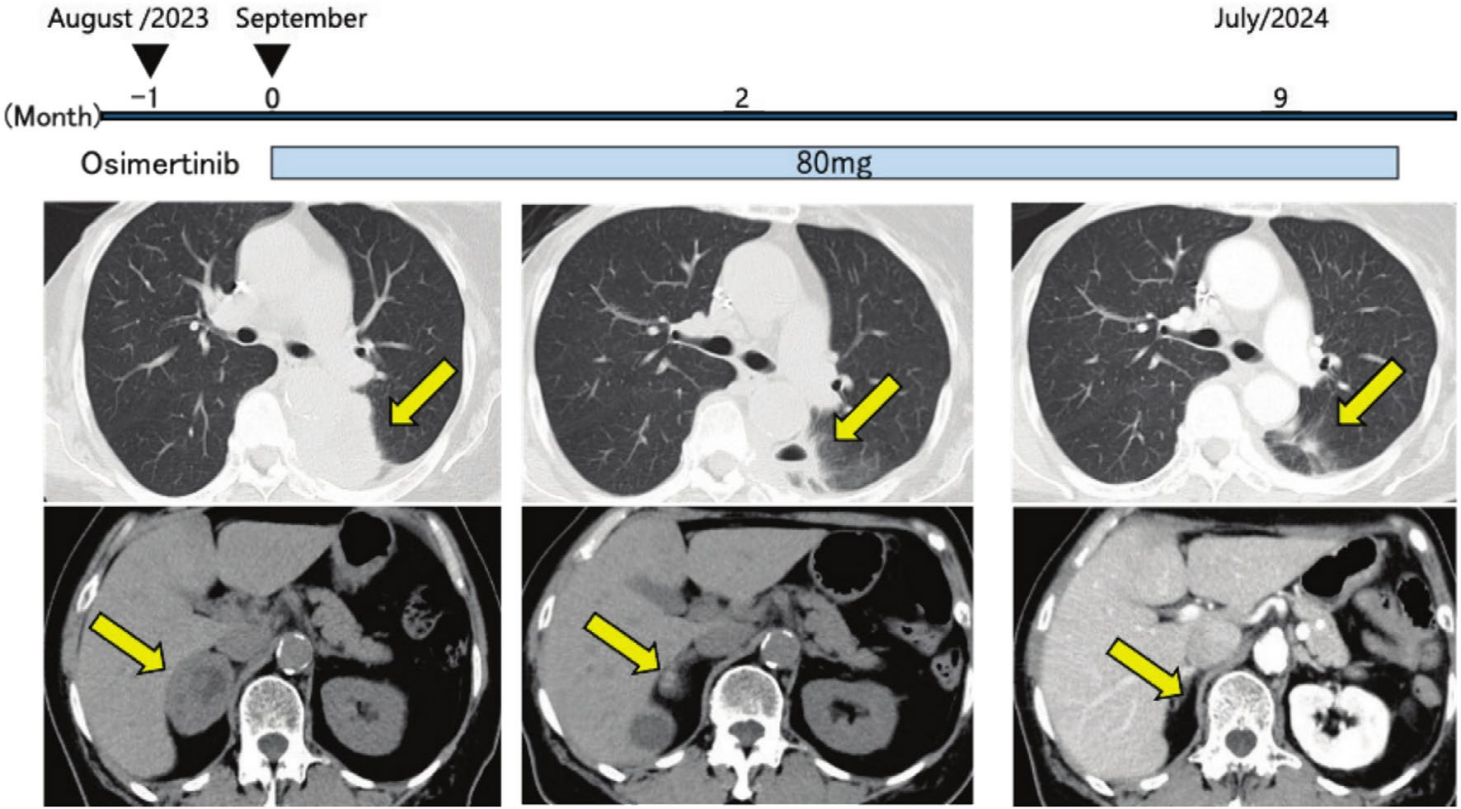
The clinical course of the patient after her referral to NHO Kochi Hospital. Osimertinib (80 mg/day) was initiated as first‐line therapy. After approximately 2 months of treatment, the size of the tumor decreased, and a partial response (PR) was achieved and sustained over 9 months of treatment. There were no remarkable adverse events. The patient showed good adherence and tolerability to osimertinib. Yellow arrows indicate the tumor.

Osimertinib (80 mg/day) was initiated as first‐line therapy. She had no remarkable adverse events and showed good adherence and tolerability to osimertinib. The size of the tumor decreased after approximately 2 months of treatment, and a partial response (PR) was achieved and sustained over 9 months of treatment (Figure 3).

## 3 Discussion

We herein presented a rare *EGFR*‐mutated squamous cell lung carcinoma that responded well to osimertinib.

A previous study reported that first‐generation EGFR‐TKIs were less effective for *EGFR*‐mutated squamous cell carcinoma than for adenocarcinoma, with an objective response rate of 31.6% and median progression‐free survival (PFS) of 3.08 months [9]. To the best of our knowledge, osimertinib has been administered as a first‐line targeted therapy to five patients with untreated *EGFR*‐mutated squamous cell lung carcinoma (Table 1) [10, 11, 12, 13]. No differences were observed based on sex or smoking history between patients with the exon 21 L858R mutation and *EGFR* exon 19 deletion mutation. The efficacy of osimertinib for *EGFR*‐mutated lung adenocarcinoma was previously shown to be excellent, with 80% of patients achieving a partial or complete response and median PFS increasing to 18.9 months [14]. In contrast, another study reported a lower response rate in *EGFR*‐mutated squamous cell lung carcinoma and shorter PFS [9]. The present case maintained PR over 9 months of treatment, the longest PFS in the literature.

**TABLE 1:** Clinical cases of *EGFR*‐mutated squamous cell lung carcinoma treated with first‐line Osimertinib.

No	Report	Age	Sex	Histological type	TTF‐1	Smoking history	EGFR mutation	PD‐L1 TPS (%)	First TKI	Response (PFS)
1	Peng [10]	50	M	SCC	Negative	Yes	Del 19 + T790M	Unknown	Osimertinib	CR (8 months)
2	Shoji [11]	63	M	SCC	Negative	Yes	L858R	Unknown	Osimertinib	PR (3 weeks)
3	Rekowska [12]	63	F	SCC	Unknown	No	L858R	Unknown	Osimertinib	PD (3 months)
4	Nishimura [13]	83	M	SCC	Negative	Yes	Del 19	< 1	Osimertinib	PD (18 days)
5	Our case	75	F	SCC	Negative	No	L858R	95	Osimertinib	PR (9 months)

Abbreviations: CR: complete response, EGFR: epidermal growth factor receptor, PD: progressive disease, PD‐L1: programmed cell death ligand 1, PFS: progression free survival, PR: partial response, SCC: squamous cell carcinoma, TKI: tyrosine kinase inhibitor, TPS: tumor proportion score, TTF‐1: thyroid transcription factor‐1.

It currently remains unclear why EGFR‐TKIs are less effective for *EGFR*‐mutated squamous cell lung carcinomas than for adenocarcinomas. The reported rate of *EGFR* mutations in patients with squamous cell lung carcinoma is 4.2%–7% [5, 6, 7, 8]. Squamous cell lung carcinomas are associated with many genomic abnormalities, and *EGFR* mutations alone may be less common. The next‐generation sequencing of tumors from patients with *EGFR*‐mutated squamous cell lung carcinoma who did not respond to osimertinib showed the amplification of *TP53, R158L, CDK6*, and *KRAS*. These co‐mutations are considered to reduce drug efficacy [13].

A previous study reported that patients who responded to first‐generation EGFR‐TKI had high serum CEA levels, TTF‐1‐positive tissue immunostaining, and were nonsmokers [15], all these findings suggestive of adenocarcinoma, indicating that patients who respond to EGFR‐TKI may also have adenocarcinoma components. Our patient had no history of smoking, her serum CEA level was within the normal range, and pathology was negative for TTF‐1 and NapsinA, strongly positive for CK5/6, and positive for p40 staining. Although we did not pathologically examine the entire tumor, adenosquamous carcinoma was unlikely. It is unclear why this patient responded to the third‐generation EGFR‐TKI. Difficulties are associated with differentiating between adenocarcinoma and squamous cell carcinoma in some cases. Our patient maintained PR over 9 months of treatment and had no remarkable adverse events. Therefore, in *EGFR*‐mutated squamous cell lung cancer, we need to consider treatments that are potentially beneficial and have few adverse events, such as TKIs [16]. The further accumulation of cases and the identification of predictive biomarkers are needed.

## 4 Conclusion

We herein presented a rare *EGFR*‐mutated squamous cell lung carcinoma that responded well to osimertinib. Osimertinib may be an option for the treatment of patients with *EGFR*‐mutated squamous cell lung carcinoma.

## Author Contributions

**Yugo Matsumura:** conceptualization, visualization, writing – original draft. **Eiji Tacheuchi:** conceptualization, writing – reviewing and editing. **Seiya Ichihara:** data curation. **Kaori Nii:** data curation. **Kazumasa Nanjo:** data curation. **Keishi Naruse:** data curation. **Naoki Kadota:** data curation. **Yoshio Okano:** data curation. **Hisanori Machida :** data curation. **Nobuo Hatakeyama :** data curation. **Hiroyuki Hino :** data curation. **Shoji Sakiyama :** data curation. **Tsutomu Shinohara :** supervision.

## Ethics Statement

The authors confirm that informed consent was obtained for the publication of this report.

## Consent

Written informed consent was obtained from the patient to publish anonymized data and accompanying images.

## Conflicts of Interest

The authors declare no conflicts of interest.

## Data Availability

Data supporting the findings of this case are available from the corresponding author upon reasonable request.
